# 4-Methyl-1,3-bis­(3,4-methyl­enedioxy­benz­yl)-2-(3,4-methyl­enedioxy­phen­yl)imidazolidine

**DOI:** 10.1107/S1600536809046662

**Published:** 2009-11-11

**Authors:** Shu-Ping Yang, Li-Jun Han, Ai-Ping Wen, Da-Qi Wang

**Affiliations:** a School of Chemical Engineering, Huaihai Institute of Technology, Lianyungang 222005, People’s Republic of China; b School of Mathematics and Science, Huaihai Institute of Technology, Lianyungang 222005, People’s Republic of China; c School of Pharmacy, Inner Mongolia Medical College, Hohhot 010059, People’s Republic of China; d College of Chemistry and Chemical Engineering, Liaocheng University, Shandong 252059, People’s Republic of China

## Abstract

In the title compound, C_27_H_26_N_2_O_6_, the imidazolidine ring adopts an envelope conformation. The methyl group on the imidazolidine ring is disordered over two positions with occupancies of 0.517 (11) and 0.483 (11), and the 3,4-methyl­enedioxy­phenyl at the 3-position of imidazolidine ring is also disordered over two positions with occupancies of 0.60 (2) and 0.40 (2).

## Related literature

For biological activity of imidazolidine derivatives, see: Sasho *et al.* (1994 ▶). For related compounds, see: Xia *et al.* (2007 ▶); Iskenderov *et al.* (2009 ▶).
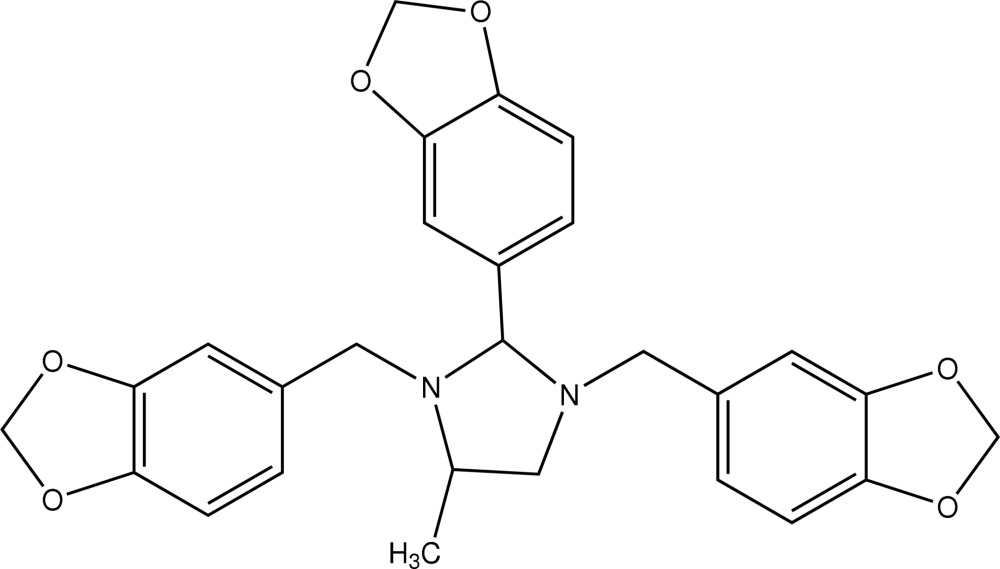


## Experimental

### 

#### Crystal data

C_27_H_26_N_2_O_6_
*M* *_r_* = 474.50
Orthorhombic, 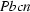

*a* = 11.586 (2) Å
*b* = 12.575 (3) Å
*c* = 32.749 (7) Å
*V* = 4771.3 (17) Å^3^
*Z* = 8
Mo *K*α radiation
μ = 0.09 mm^−1^
*T* = 298 K
0.50 × 0.40 × 0.30 mm

#### Data collection

Bruker SMART CCD area-detector diffractometer
Absorption correction: multi-scan (*SADABS*; Sheldrick, 1996 ▶) *T*_min_ = 0.955, *T*_max_ = 0.972
19793 measured reflections
4200 independent reflections
2896 reflections with *I* > 2σ(*I*)
*R*_int_ = 0.055

#### Refinement

*R*[*F* ^2^ > 2σ(*F* ^2^)] = 0.066
*wR*(*F* ^2^) = 0.215
*S* = 1.07
4200 reflections
392 parameters
H-atom parameters constrained
Δρ_max_ = 0.40 e Å^−3^
Δρ_min_ = −0.43 e Å^−3^

### 

Data collection: *SMART* (Siemens, 1996 ▶); cell refinement: *SAINT* (Siemens, 1996 ▶); data reduction: *SAINT*; program(s) used to solve structure: *SHELXTL* (Sheldrick, 2008 ▶); program(s) used to refine structure: *SHELXTL*; molecular graphics: *SHELXTL*; software used to prepare material for publication: *SHELXTL*.

## Supplementary Material

Crystal structure: contains datablocks I, global. DOI: 10.1107/S1600536809046662/xu2638sup1.cif

Structure factors: contains datablocks I. DOI: 10.1107/S1600536809046662/xu2638Isup2.hkl

Additional supplementary materials:  crystallographic information; 3D view; checkCIF report

## Figures and Tables

**Fig. 1.**
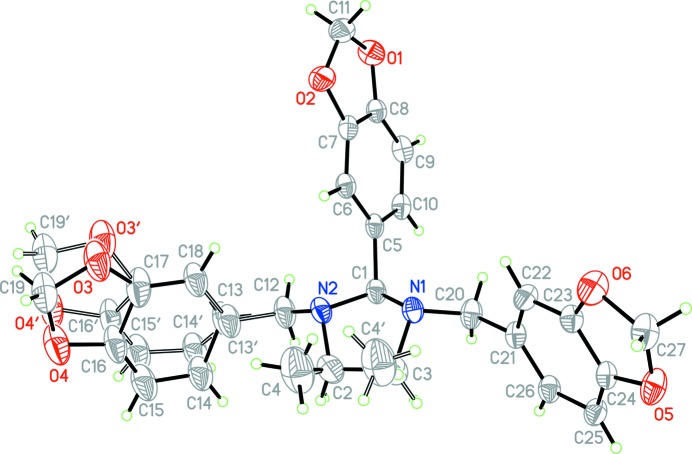
The molecular structure of (I), showing the disorder components and the atom-labelling scheme. Displacement ellipsoids are drawn at the 30% probability level.

## supplementary crystallographic information

### Comment

Some imidazolidine derivatives exhibit a broad spectrum of biological activities (Sasho *et al.*, 1994). The crystal structures of some imidazolidine compounds have reported (Xia *et al.*, 2007; Iskenderov *et al.*, 2009). Here, we report the crystal structure of a new imidazolidine compound (I).

In the title compound (I) (Fig.1), the imidazolidine ring adopts an envelope conformation. The methyl on the imidazolidine ring is disordered over two positions, leading to positional disorder of the two methylene H atoms of the ring, with occupancies of 0.517 (11) and 0.483 (11).

The 3, 4-methylenedioxyphenyl at the 3-position on imidazolidine ring is disordered over two positions, with occupancies of 0.60 (2) and 0.40 (2). The dihedral angles between the planes of the two disordered benzyl rings C13—C18 and C13'-C16'/C17/C18 is 26 (7)°, and the planes of the two disordered methylenedioxy rings C17/O3/C19/O4/C16 and C17/O3'/C19'/O4'/C16' are 21 (1)°.

In the imidazolidine ring, the C—N bond lengths range from 1.435 (4) to 1.542 (4) Å, which are close the average single C—N bond lengths of 1.48 Å.

### Experimental

The reaction mixture containing 3,4-methylenedioxybenzaldehyde (3.0 g, 20 mmol) and propane-1,2-diamine (2.8 g, 20 mmol) was refluxed for about 4 h in ethanol (30 ml), then the borohydride sodium (1.52 g, 40 mmol) was added and stirred for 4 h (at 333–343 K), and the reaction mixture was cooled and the crude products were filtered off, washed with water and ethanol, then dried. Colourless crystals of (I) suitable for X-ray structure analysis were obtained by recrystallizing the crude product from ethanol (m.p. 478–480 K).

### Refinement

Methyl group on imidazolidine ring is disorder, and the 3,4-methylenedioxyphenyl at the 3-position on imidazolidine ring is also disorder. The site occupancies of the methyl group were refined to 0.517 (11) and 0.483 (11), and the site occupancies of the 3,4-methylenedioxyphenyl were refined to 0.60 (2) and 0.40 (2).

H atoms were placed in calculated positions with C—H = 0.93 (aromatic), 0.97 (methylene) and 0.96 Å (methyl), and refined in riding mode with *U*_iso_(H) = 1.5*U*_eq_(C) for methyl H atoms and 1.2*U*_eq_(C) for the others.

### Crystal data

**Table:**

C_27_H_26_N_2_O_6_	*D*_x_ = 1.321 Mg m^−^^3^
*M**_r_* = 474.50	Melting point: 478 K
Orthorhombic, *P**b**c**n*	Mo *K*α radiation, λ = 0.71073 Å
Hall symbol: -P 2n 2ab	Cell parameters from 4200 reflections
*a* = 11.586 (2) Å	θ = 1.2–25.0°
*b* = 12.575 (3) Å	µ = 0.09 mm^−^^1^
*c* = 32.749 (7) Å	*T* = 298 K
*V* = 4771.3 (17) Å^3^	Block, yellow
*Z* = 8	0.50 × 0.40 × 0.30 mm
*F*(000) = 2000	

### Data collection

**Table:**

Bruker SMART CCD area-detector diffractometer	4200 independent reflections
Radiation source: fine-focus sealed tube	2896 reflections with *I* > 2σ(*I*)
graphite	*R*_int_ = 0.055
φ and ω scans	θ_max_ = 25.0°, θ_min_ = 1.2°
Absorption correction: multi-scan (*SADABS*; Sheldrick, 1996)	*h* = −13→12
*T*_min_ = 0.955, *T*_max_ = 0.972	*k* = −10→14
19793 measured reflections	*l* = −35→38

### Refinement

**Table:**

Refinement on *F*^2^	Primary atom site location: structure-invariant direct methods
Least-squares matrix: full	Secondary atom site location: difference Fourier map
*R*[*F*^2^ > 2σ(*F*^2^)] = 0.066	Hydrogen site location: inferred from neighbouring sites
*wR*(*F*^2^) = 0.215	H-atom parameters constrained
*S* = 1.07	*w* = 1/[σ^2^(*F*_o_^2^) + (0.1256*P*)^2^ + 1.4629*P*] where *P* = (*F*_o_^2^ + 2*F*_c_^2^)/3
4200 reflections	(Δ/σ)_max_ < 0.001
392 parameters	Δρ_max_ = 0.40 e Å^−^^3^
0 restraints	Δρ_min_ = −0.43 e Å^−^^3^

### Special details

**Table:**

Geometry. All e.s.d.'s (except the e.s.d. in the dihedral angle between two l.s. planes) are estimated using the full covariance matrix. The cell e.s.d.'s are taken into account individually in the estimation of e.s.d.'s in distances, angles and torsion angles; correlations between e.s.d.'s in cell parameters are only used when they are defined by crystal symmetry. An approximate (isotropic) treatment of cell e.s.d.'s is used for estimating e.s.d.'s involving l.s. planes.
Refinement. Refinement of *F*^2^ against ALL reflections. The weighted *R*-factor *wR* and goodness of fit *S* are based on *F*^2^, conventional *R*-factors *R* are based on *F*, with *F* set to zero for negative *F*^2^. The threshold expression of *F*^2^ > σ(*F*^2^) is used only for calculating *R*-factors(gt) *etc*. and is not relevant to the choice of reflections for refinement. *R*-factors based on *F*^2^ are statistically about twice as large as those based on *F*, and *R*- factors based on ALL data will be even larger.

### Fractional atomic coordinates and isotropic or equivalent isotropic displacement parameters (Å^2^)

**Table:**

	*x*	*y*	*z*	*U*_iso_*/*U*_eq_	Occ. (<1)
N1	0.54794 (18)	0.07206 (19)	0.38949 (7)	0.0516 (6)	
N2	0.65433 (19)	0.1297 (2)	0.33661 (7)	0.0560 (6)	
O1	0.57024 (18)	0.60432 (18)	0.42342 (7)	0.0681 (6)	
O2	0.48948 (18)	0.53485 (17)	0.36519 (6)	0.0675 (6)	
O3	0.6527 (14)	0.3038 (15)	0.1842 (4)	0.103 (4)	0.60 (2)
O4	0.7802 (7)	0.1936 (14)	0.1522 (2)	0.111 (4)	0.60 (2)
O3'	0.671 (2)	0.346 (2)	0.1934 (7)	0.108 (6)	0.40 (2)
O4'	0.8033 (11)	0.2699 (19)	0.1568 (3)	0.105 (5)	0.40 (2)
O5	0.18733 (17)	−0.21867 (19)	0.47927 (7)	0.0725 (7)	
O6	0.15747 (17)	−0.03598 (19)	0.45358 (8)	0.0790 (7)	
C1	0.6424 (2)	0.1424 (2)	0.38092 (8)	0.0505 (7)	
H1	0.7120	0.1146	0.3941	0.061*	
C2	0.6315 (4)	0.0114 (3)	0.32760 (12)	0.0877 (12)	
H2	0.7018	−0.0304	0.3232	0.105*	0.517 (11)
H2A	0.5912	0.0032	0.3018	0.105*	0.483 (11)
H2B	0.7035	−0.0278	0.3263	0.105*	0.483 (11)
C3	0.5571 (3)	−0.0288 (3)	0.36290 (12)	0.0814 (11)	
H3A	0.5946	−0.0864	0.3775	0.098*	0.517 (11)
H3B	0.4819	−0.0523	0.3535	0.098*	0.517 (11)
H3	0.5801	−0.0952	0.3763	0.098*	0.483 (11)
C4	0.5461 (10)	0.0063 (10)	0.2920 (3)	0.137 (5)	0.517 (11)
H4A	0.5292	−0.0667	0.2858	0.206*	0.517 (11)
H4B	0.5793	0.0400	0.2685	0.206*	0.517 (11)
H4C	0.4761	0.0423	0.2994	0.206*	0.517 (11)
C4'	0.4468 (10)	−0.0296 (10)	0.3372 (3)	0.137 (5)	0.483 (11)
H4'1	0.4419	0.0352	0.3218	0.206*	0.483 (11)
H4'2	0.3809	−0.0354	0.3548	0.206*	0.483 (11)
H4'3	0.4485	−0.0890	0.3188	0.206*	0.483 (11)
C5	0.6280 (2)	0.2673 (2)	0.39295 (8)	0.0452 (7)	
C6	0.5621 (2)	0.3386 (2)	0.36982 (8)	0.0485 (7)	
H6	0.5268	0.3156	0.3459	0.058*	
C7	0.5486 (2)	0.4488 (2)	0.38306 (8)	0.0491 (7)	
C8	0.5974 (2)	0.4909 (2)	0.41752 (8)	0.0491 (7)	
C9	0.6641 (2)	0.4241 (3)	0.44031 (9)	0.0559 (8)	
H9	0.7001	0.4489	0.4638	0.067*	
C10	0.6793 (2)	0.3109 (2)	0.42730 (8)	0.0504 (7)	
H10	0.7258	0.2672	0.4432	0.060*	
C11	0.4924 (3)	0.6290 (3)	0.39238 (10)	0.0693 (9)	
H11A	0.5178	0.6915	0.3775	0.083*	
H11B	0.4164	0.6429	0.4036	0.083*	
C12	0.7609 (3)	0.1641 (3)	0.32329 (10)	0.0744 (10)	
H12A	0.8188	0.1132	0.3320	0.089*	
H12B	0.7783	0.2314	0.3364	0.089*	
C13	0.771 (16)	0.18 (2)	0.276 (5)	0.078 (13)	0.60 (2)
C14	0.8314 (9)	0.0937 (14)	0.2552 (4)	0.090 (3)	0.60 (2)
H14	0.8619	0.0364	0.2695	0.108*	0.60 (2)
C15	0.8420 (10)	0.1013 (14)	0.2130 (3)	0.099 (4)	0.60 (2)
H15	0.8895	0.0548	0.1987	0.119*	0.60 (2)
C16	0.7852 (14)	0.1733 (16)	0.1943 (5)	0.093 (4)	0.60 (2)
C13'	0.77 (2)	0.17 (3)	0.280 (8)	0.08 (2)	0.40 (2)
C14'	0.8675 (17)	0.150 (2)	0.2597 (5)	0.090 (5)	0.40 (2)
H14'	0.9277	0.1201	0.2746	0.108*	0.40 (2)
C15'	0.8866 (16)	0.171 (2)	0.2187 (4)	0.101 (6)	0.40 (2)
H15'	0.9528	0.1466	0.2057	0.121*	0.40 (2)
C16'	0.804 (2)	0.231 (2)	0.1972 (7)	0.093 (7)	0.40 (2)
C17	0.7175 (3)	0.2566 (4)	0.21498 (12)	0.0983 (14)	
C18	0.7029 (3)	0.2470 (4)	0.25621 (10)	0.0825 (11)	
H18	0.6475	0.2867	0.2700	0.099*	
C19	0.7006 (9)	0.2752 (16)	0.1463 (3)	0.106 (4)	0.60 (2)
H19A	0.6400	0.2516	0.1279	0.128*	0.60 (2)
H19B	0.7384	0.3362	0.1341	0.128*	0.60 (2)
C19'	0.7263 (14)	0.356 (2)	0.1554 (5)	0.106 (6)	0.40 (2)
H19C	0.7669	0.4236	0.1531	0.128*	0.40 (2)
H19D	0.6721	0.3497	0.1329	0.128*	0.40 (2)
C20	0.5440 (2)	0.0408 (3)	0.43233 (9)	0.0621 (8)	
H20A	0.5367	0.1045	0.4488	0.075*	
H20B	0.6172	0.0078	0.4393	0.075*	
C21	0.4498 (2)	−0.0335 (2)	0.44382 (8)	0.0499 (7)	
C22	0.3463 (2)	0.0101 (2)	0.44082 (9)	0.0559 (7)	
H22	0.3315	0.0787	0.4316	0.067*	
C23	0.2654 (2)	−0.0602 (2)	0.45320 (8)	0.0513 (7)	
C24	0.2828 (2)	−0.1680 (2)	0.46835 (8)	0.0487 (7)	
C25	0.3817 (2)	−0.2133 (2)	0.47131 (9)	0.0574 (8)	
H25	0.3948	−0.2824	0.4804	0.069*	
C26	0.4654 (2)	−0.1425 (2)	0.45883 (9)	0.0558 (8)	
H26	0.5412	−0.1667	0.4601	0.067*	
C27	0.1083 (3)	−0.1372 (3)	0.46922 (11)	0.0717 (9)	
H27A	0.0632	−0.1212	0.4934	0.086*	
H27B	0.0555	−0.1648	0.4488	0.086*	

### Atomic displacement parameters (Å^2^)

**Table:**

	*U*^11^	*U*^22^	*U*^33^	*U*^12^	*U*^13^	*U*^23^
N1	0.0434 (12)	0.0635 (15)	0.0478 (13)	−0.0051 (11)	0.0046 (10)	0.0013 (11)
N2	0.0473 (12)	0.0744 (17)	0.0464 (13)	0.0051 (11)	0.0106 (10)	−0.0002 (11)
O1	0.0632 (12)	0.0731 (15)	0.0681 (14)	0.0019 (11)	−0.0056 (10)	−0.0107 (11)
O2	0.0709 (13)	0.0721 (15)	0.0594 (13)	0.0163 (11)	−0.0148 (10)	0.0034 (11)
O3	0.082 (5)	0.177 (12)	0.051 (5)	0.026 (6)	−0.014 (4)	0.002 (5)
O4	0.086 (4)	0.186 (10)	0.061 (3)	0.029 (5)	0.013 (3)	0.001 (4)
O3'	0.087 (9)	0.178 (19)	0.059 (10)	0.017 (10)	−0.007 (7)	0.016 (9)
O4'	0.089 (6)	0.170 (14)	0.056 (5)	0.019 (8)	0.022 (4)	0.018 (7)
O5	0.0477 (11)	0.0909 (17)	0.0790 (16)	−0.0153 (11)	−0.0004 (10)	0.0278 (12)
O6	0.0392 (11)	0.0932 (18)	0.1048 (19)	0.0071 (11)	0.0014 (11)	0.0294 (14)
C1	0.0401 (13)	0.0655 (19)	0.0459 (15)	0.0011 (12)	0.0079 (11)	0.0053 (13)
C2	0.106 (3)	0.083 (3)	0.074 (2)	−0.003 (2)	0.025 (2)	−0.011 (2)
C3	0.078 (2)	0.084 (3)	0.082 (2)	−0.0179 (19)	0.0173 (19)	−0.016 (2)
C4	0.142 (9)	0.179 (11)	0.092 (7)	−0.063 (8)	0.002 (6)	−0.025 (7)
C4'	0.142 (10)	0.179 (12)	0.092 (7)	−0.063 (8)	0.002 (6)	−0.025 (7)
C5	0.0328 (12)	0.0638 (18)	0.0391 (14)	−0.0030 (12)	0.0048 (10)	0.0073 (12)
C6	0.0421 (13)	0.0663 (18)	0.0369 (13)	−0.0011 (13)	−0.0017 (11)	0.0014 (13)
C7	0.0412 (13)	0.0646 (19)	0.0416 (14)	0.0006 (13)	0.0015 (11)	0.0098 (13)
C8	0.0388 (13)	0.0606 (18)	0.0479 (15)	−0.0056 (12)	0.0043 (12)	0.0011 (13)
C9	0.0409 (13)	0.081 (2)	0.0454 (15)	−0.0108 (14)	−0.0040 (12)	−0.0026 (14)
C10	0.0322 (12)	0.076 (2)	0.0426 (15)	−0.0022 (12)	−0.0012 (11)	0.0099 (14)
C11	0.072 (2)	0.069 (2)	0.067 (2)	0.0129 (17)	−0.0043 (17)	0.0012 (17)
C12	0.0470 (16)	0.122 (3)	0.055 (2)	0.0127 (17)	0.0109 (15)	0.0133 (18)
C13	0.052 (9)	0.13 (3)	0.050 (15)	0.020 (14)	0.022 (9)	0.018 (14)
C14	0.063 (5)	0.137 (10)	0.071 (5)	0.021 (5)	0.023 (4)	0.012 (6)
C15	0.070 (5)	0.160 (11)	0.068 (5)	0.030 (6)	0.020 (4)	0.000 (6)
C16	0.065 (6)	0.159 (13)	0.054 (5)	0.015 (8)	0.021 (4)	0.007 (8)
C13'	0.06 (4)	0.13 (6)	0.06 (6)	0.02 (3)	0.01 (4)	0.01 (5)
C14'	0.062 (9)	0.144 (16)	0.063 (7)	0.033 (9)	0.014 (6)	0.011 (9)
C15'	0.075 (9)	0.162 (16)	0.067 (7)	0.026 (10)	0.020 (6)	0.012 (9)
C16'	0.071 (10)	0.16 (2)	0.051 (8)	0.022 (12)	0.020 (7)	0.019 (12)
C17	0.058 (2)	0.173 (4)	0.064 (2)	0.023 (2)	0.0014 (18)	0.030 (3)
C18	0.0564 (19)	0.136 (3)	0.055 (2)	0.022 (2)	0.0081 (16)	0.007 (2)
C19	0.084 (6)	0.175 (13)	0.060 (5)	0.021 (7)	0.007 (4)	0.015 (6)
C19'	0.084 (8)	0.175 (18)	0.060 (7)	0.021 (10)	0.007 (6)	0.015 (9)
C20	0.0460 (15)	0.087 (2)	0.0538 (17)	−0.0097 (14)	−0.0003 (13)	0.0143 (16)
C21	0.0422 (14)	0.0650 (19)	0.0426 (15)	−0.0049 (13)	0.0007 (11)	0.0061 (13)
C22	0.0508 (16)	0.0562 (18)	0.0608 (18)	−0.0002 (13)	−0.0001 (14)	0.0141 (14)
C23	0.0396 (14)	0.0665 (19)	0.0479 (16)	0.0013 (13)	−0.0008 (12)	0.0061 (13)
C24	0.0442 (14)	0.0637 (18)	0.0381 (14)	−0.0058 (13)	−0.0001 (11)	0.0066 (12)
C25	0.0535 (16)	0.0630 (19)	0.0558 (17)	0.0047 (14)	0.0033 (13)	0.0163 (14)
C26	0.0448 (14)	0.072 (2)	0.0512 (16)	0.0047 (13)	0.0032 (12)	0.0107 (14)
C27	0.0446 (16)	0.101 (3)	0.070 (2)	−0.0090 (17)	−0.0053 (15)	0.0166 (19)

### Geometric parameters (Å, °)

**Table:**

N1—C1	1.435 (3)	C8—C9	1.363 (4)
N1—C20	1.458 (4)	C9—C10	1.497 (4)
N1—C3	1.542 (4)	C9—H9	0.9300
N2—C12	1.379 (4)	C10—H10	0.9300
N2—C1	1.467 (4)	C11—H11A	0.9700
N2—C2	1.541 (4)	C11—H11B	0.9700
O1—C11	1.395 (4)	C12—C13'	1.4 (3)
O1—C8	1.474 (4)	C12—C13	1.57 (17)
O2—C7	1.408 (3)	C12—H12A	0.9700
O2—C11	1.482 (4)	C12—H12B	0.9700
O3—C17	1.389 (17)	C13—C18	1.3 (2)
O3—C19	1.409 (17)	C13—C14	1.4 (3)
O4—C19	1.394 (13)	C14—C15	1.392 (14)
O4—C16	1.402 (18)	C14—H14	0.9300
O3'—C19'	1.40 (3)	C15—C16	1.277 (19)
O3'—C17	1.43 (3)	C15—H15	0.9300
O4'—C19'	1.407 (19)	C16—C17	1.474 (18)
O4'—C16'	1.41 (3)	C13'—C14'	1.4 (3)
O5—C24	1.325 (3)	C13'—C18	1.4 (4)
O5—C27	1.413 (4)	C14'—C15'	1.39 (2)
O6—C23	1.288 (3)	C14'—H14'	0.9300
O6—C27	1.486 (4)	C15'—C16'	1.41 (3)
C1—C5	1.628 (4)	C15'—H15'	0.9300
C1—H1	0.9800	C16'—C17	1.20 (2)
C2—C3	1.528 (5)	C17—C18	1.367 (5)
C2—C4	1.532 (11)	C18—H18	0.9300
C2—H2	0.9800	C19—H19A	0.9700
C2—H2A	0.9700	C19—H19B	0.9700
C2—H2B	0.9700	C19'—H19C	0.9700
C3—C4'	1.531 (12)	C19'—H19D	0.9700
C3—H3A	0.9700	C20—C21	1.485 (4)
C3—H3B	0.9700	C20—H20A	0.9700
C3—H3	0.9800	C20—H20B	0.9700
C4—H4A	0.9600	C21—C22	1.322 (4)
C4—H4B	0.9600	C21—C26	1.467 (4)
C4—H4C	0.9600	C22—C23	1.351 (4)
C4'—H4'1	0.9600	C22—H22	0.9300
C4'—H4'2	0.9600	C23—C24	1.458 (4)
C4'—H4'3	0.9600	C24—C25	1.284 (4)
C5—C10	1.385 (4)	C25—C26	1.379 (4)
C5—C6	1.400 (4)	C25—H25	0.9300
C6—C7	1.461 (4)	C26—H26	0.9300
C6—H6	0.9300	C27—H27A	0.9700
C7—C8	1.369 (4)	C27—H27B	0.9700
			
C1—N1—C20	112.2 (2)	N2—C12—H12B	108.6
C1—N1—C3	110.1 (2)	C13'—C12—H12B	113.0
C20—N1—C3	108.9 (3)	C13—C12—H12B	108.6
C12—N2—C1	111.3 (2)	H12A—C12—H12B	107.6
C12—N2—C2	113.3 (3)	C18—C13—C14	122 (10)
C1—N2—C2	106.1 (2)	C18—C13—C12	121 (10)
C11—O1—C8	105.0 (2)	C14—C13—C12	114 (10)
C7—O2—C11	110.7 (2)	C15—C14—C13	117 (8)
C17—O3—C19	108.5 (12)	C15—C14—H14	121.4
C19—O4—C16	107.3 (9)	C13—C14—H14	121.4
C19'—O3'—C17	110.1 (18)	C16—C15—C14	118.7 (11)
C19'—O4'—C16'	107.5 (13)	C16—C15—H15	120.6
C24—O5—C27	97.4 (2)	C14—C15—H15	120.6
C23—O6—C27	100.0 (2)	C15—C16—O4	128.5 (13)
N1—C1—N2	101.5 (2)	C15—C16—C17	123.9 (13)
N1—C1—C5	118.0 (2)	O4—C16—C17	107.5 (12)
N2—C1—C5	110.7 (2)	C14'—C13'—C12	123 (10)
N1—C1—H1	108.8	C14'—C13'—C18	107 (10)
N2—C1—H1	108.8	C12—C13'—C18	123 (10)
C5—C1—H1	108.8	C13'—C14'—C15'	125 (10)
C3—C2—C4	101.4 (5)	C13'—C14'—H14'	117.7
C3—C2—N2	105.8 (3)	C15'—C14'—H14'	117.7
C4—C2—N2	107.3 (6)	C14'—C15'—C16'	118.5 (14)
C3—C2—H2	113.8	C14'—C15'—H15'	120.7
C4—C2—H2	113.8	C16'—C15'—H15'	120.7
N2—C2—H2	113.8	C17—C16'—C15'	118.0 (19)
C3—C2—H2A	110.6	C17—C16'—O4'	111 (2)
N2—C2—H2A	110.6	C15'—C16'—O4'	130.9 (19)
H2—C2—H2A	102.5	C16'—C17—C18	124.0 (13)
C3—C2—H2B	110.6	C16'—C17—O3	102.3 (14)
C4—C2—H2B	120.0	C18—C17—O3	133.4 (7)
N2—C2—H2B	110.6	C16'—C17—O3'	106.3 (16)
H2A—C2—H2B	108.7	C18—C17—O3'	120.8 (10)
C2—C3—C4'	93.2 (5)	C18—C17—C16	117.3 (8)
C2—C3—N1	101.2 (3)	O3—C17—C16	104.9 (9)
C4'—C3—N1	105.0 (6)	O3'—C17—C16	121.9 (12)
C2—C3—H3A	111.5	C13—C18—C17	118 (8)
C4'—C3—H3A	129.8	C17—C18—C13'	122 (10)
N1—C3—H3A	111.5	C13—C18—H18	121.2
C2—C3—H3B	111.5	C17—C18—H18	121.2
N1—C3—H3B	111.5	C13'—C18—H18	118.0
H3A—C3—H3B	109.3	O4—C19—O3	109.0 (10)
C2—C3—H3	117.9	O4—C19—H19A	109.9
C4'—C3—H3	117.9	O3—C19—H19A	109.9
N1—C3—H3	117.9	O4—C19—H19B	109.9
C2—C4—H4A	109.5	O3—C19—H19B	109.9
C2—C4—H4B	109.5	H19A—C19—H19B	108.3
C2—C4—H4C	109.5	O3'—C19'—O4'	100.6 (15)
C3—C4'—H4'1	109.5	O3'—C19'—H19C	111.6
C3—C4'—H4'2	109.5	O4'—C19'—H19C	111.6
H4'1—C4'—H4'2	109.5	O3'—C19'—H19D	111.6
C3—C4'—H4'3	109.5	O4'—C19'—H19D	111.6
H4'1—C4'—H4'3	109.5	H19C—C19'—H19D	109.4
H4'2—C4'—H4'3	109.5	N1—C20—C21	115.9 (2)
C10—C5—C6	114.8 (3)	N1—C20—H20A	108.3
C10—C5—C1	122.3 (2)	C21—C20—H20A	108.3
C6—C5—C1	122.8 (2)	N1—C20—H20B	108.3
C5—C6—C7	120.4 (2)	C21—C20—H20B	108.3
C5—C6—H6	119.8	H20A—C20—H20B	107.4
C7—C6—H6	119.8	C22—C21—C26	121.6 (3)
C8—C7—O2	104.3 (2)	C22—C21—C20	112.8 (3)
C8—C7—C6	124.5 (2)	C26—C21—C20	125.5 (2)
O2—C7—C6	131.2 (2)	C21—C22—C23	109.6 (3)
C9—C8—C7	116.6 (3)	C21—C22—H22	125.2
C9—C8—O1	130.2 (3)	C23—C22—H22	125.2
C7—C8—O1	113.2 (2)	O6—C23—C22	121.5 (3)
C8—C9—C10	119.7 (2)	O6—C23—C24	110.5 (2)
C8—C9—H9	120.1	C22—C23—C24	128.0 (2)
C10—C9—H9	120.1	C25—C24—O5	120.8 (3)
C5—C10—C9	123.9 (2)	C25—C24—C23	124.1 (3)
C5—C10—H10	118.1	O5—C24—C23	115.1 (2)
C9—C10—H10	118.1	C24—C25—C26	108.6 (3)
O1—C11—O2	105.9 (2)	C24—C25—H25	125.7
O1—C11—H11A	110.5	C26—C25—H25	125.7
O2—C11—H11A	110.5	C25—C26—C21	128.0 (3)
O1—C11—H11B	110.5	C25—C26—H26	116.0
O2—C11—H11B	110.5	C21—C26—H26	116.0
H11A—C11—H11B	108.7	O5—C27—O6	116.9 (2)
N2—C12—C13'	111 (10)	O5—C27—H27A	108.1
N2—C12—C13	115 (7)	O6—C27—H27A	108.1
N2—C12—H12A	108.6	O5—C27—H27B	108.1
C13'—C12—H12A	106.0	O6—C27—H27B	108.1
C13—C12—H12A	108.6	H27A—C27—H27B	107.3
